# Protocol for a randomised controlled trial of a co-produced, complex, health promotion intervention for women with prior gestational diabetes and their families: the Face-it study

**DOI:** 10.1186/s13063-020-4062-4

**Published:** 2020-02-07

**Authors:** Karoline Kragelund Nielsen, Inger Katrine Dahl-Petersen, Dorte Møller Jensen, Per Ovesen, Peter Damm, Nanna Husted Jensen, Maja Thøgersen, Anne Timm, Line Hillersdal, Ulla Kampmann, Christina Anne Vinter, Elisabeth Reinhardt Mathiesen, Helle Terkildsen Maindal, On behalf of the Face-it Study Group

**Affiliations:** 1 Health Promotion, Steno Diabetes Center Copenhagen, Niels Steensens Vej 6, 2820 Gentofte, Denmark; 2 Steno Diabetes Center Odense, Odense, Denmark; 3 Department of Gynecology and Obstetrics, Odense University Hospital, Odense, Denmark; 4 Department of Clinical Research, Faculty of Health Sciences, University of Southern Denmark, Odense, Denmark; 5 Department of Obstetrics, Aarhus University Hospital, Aarhus, Denmark; 6 Centre for Pregnant Women with Diabetes, Department of Obstetrics, Rigshospitalet, Copenhagen, Denmark; 7 Department of Clinical Medicine, University of Copenhagen, Copenhagen, Denmark; 8 Department of Public Health, Aarhus University, Aarhus, Denmark; 9 Department of Anthropology, University of Copenhagen, Copenhagen, Denmark; 10 Steno Diabetes Center Aarhus, Aarhus, Denmark; 11 Centre for Pregnant Women with Diabetes, Department of Endocrinology, Rigshospitalet, Copenhagen, Denmark

**Keywords:** Gestational diabetes mellitus, Type 2 diabetes prevention, Postpartum period, Family intervention, Complex intervention, Health promotion, Cross-disciplinary research

## Abstract

**Background:**

Gestational diabetes mellitus (GDM) is associated with an increased risk of future diabetes in both mother, father and offspring. More knowledge is needed about how to effectively reduce the risk of diabetes through sustained behavioural interventions in these families. The Face-it intervention is a complex health promotion intervention embedded in multi-level supportive environments. The aim of the intervention is to reduce type 2 diabetes risk and increase quality of life among families in the first year following a GDM-affected pregnancy by promoting physical activity, healthy dietary behaviours and breastfeeding through a focus on social support, motivation, self-efficacy, risk perception and health literacy.

**Methods:**

This national multicentre study is a two-arm randomised controlled trial including 460 women with GDM in a ratio of 2 (intervention):1 (usual care). The Face-it intervention consists of three main components: 1) additional visits from municipal health visitors, 2) digital health coaching tailored to family needs and 3) a structured cross-sectoral communication system in the health care system. The intervention runs from 3 to 12 months after delivery. The primary outcome is maternal body mass index at 12 months after delivery as a proxy for diabetes risk.

The women will be examined at baseline and at follow-up, and this examination will include blood tests, oral glucose tolerance test (OGTT), anthropometrics, blood pressure, self-reported diet and physical activity, breastfeeding, quality of life, health literacy, physical and mental health status, risk perception and social support. Aside from those data collected for OGTT and breastfeeding and offspring parameters, the same data will be collected for partners. Data on offspring anthropometry will also be collected. Information on pregnancy- and birth-related outcomes will be derived from the medical records of the woman and child.

**Discussion:**

This randomised controlled trial seeks to demonstrate whether the Face-it intervention, addressing the individual, family and health care system levels, is superior to usual care in reducing diabetes risk for mothers and their families. Coupled with a process evaluation and an economic analysis, the study will provide evidence for policymakers and health services about health promotion among families affected by GDM and the potential for reducing risk of type 2 diabetes and associated conditions.

**Trial registration:**

ClinicalTrials.gov NCT03997773. Registered June 25, 2019 – Retrospectively registered.

## Introduction

Gestational diabetes mellitus (GDM) predisposes women and their offspring to a range of short- and long-term morbidities, including early onset type 2 diabetes (T2D) and cardiovascular disease [1–5]. With a prevalence of approximately 3% in Denmark [6], equivalent to an estimated 2500 affected pregnancies annually, GDM is one of the most common conditions during pregnancy.

Evidence from the US Diabetes Prevention Program (DPP) suggests that intensive lifestyle interventions can reduce the risk of T2D among women with prior GDM [7]. However, study participants in the DPP sub-group analysis were, on average, 12 years past their last GDM-affected pregnancy [7]. Since the cumulative incidence of T2D in women with prior GDM increases substantially within the first 5 years after delivery [8], the need exists to identify effective interventions in this time period. Importantly, a recent meta-analysis of lifestyle interventions for the prevention of T2D in women with previous GDM showed that the effects appear to be larger when the intervention is initiated within 6 months after delivery [9]. However, the authors concluded that there is a need to further tailor interventions to this specific period of life and that additional studies are required to improve the quality of the evidence [9]. A 2017 systematic review also reported that while any intervention was superior to no intervention, based on the current evidence, concluding which behavioural intervention components would be the most effective in reducing T2D risk in women with prior GDM [10] was not possible.

Previous research shows that, in everyday real-life settings, changes in health behaviour are difficult to sustain. Due to a number of barriers [11, 12], many women with prior GDM do not follow recommendations for healthy diet and physical activity after delivery [13]. These barriers have been suggested to act at three levels: i) the individual level (e.g., diabetes beliefs), ii) the social/family level (e.g., social support) and iii) the health system level (e.g., poor follow-up) [14, 15]. Importantly, as the barriers tend to be interlinked and interacting, they need to be addressed in a comprehensive and multifaceted way. Thus, health promotion efforts must be based on a thorough understanding of the barriers to healthy behaviours and involve carefully tailored solutions to overcome these barriers.

Hawkins et al. proposed a framework for the development of complex interventions that facilitates adoption and maximises implementation [16]. The key features of the framework are the use of comprehensive evidence review, co-production and prototyping. Involving the target group in the development of the intervention ensures ownership and relevance and thus holds significant promise for safeguarding adoption and sustainability among women with prior GDM.

As many of the barriers to behavioural intervention are beyond the control of individual women, a consideration of the target group in less narrow terms is important [14, 17]. Focusing on the family rather than the individual is relevant as studies have shown offspring and partners of women with GDM face elevated diabetes risk themselves [4, 18]. Furthermore, behaviours tend to cluster and are patterned by the social and economic factors at play within a family household [19, 20].

This paper describes the study protocol for the Face-it randomised controlled trial (RCT), which seeks to prevent T2D and increase the quality of life in women with prior GDM and their families by the promotion of physical activity, healthy dietary behaviours and breastfeeding through a focus on social support, motivation, self-efficacy, risk perception and health literacy. The intervention is multi-level and has been developed using co-production techniques. Based on existing literature [21–24], behavioural theories [25, 26] and our comprehensive intervention development work, we hypothesise that a complex health promotion intervention with several interacting components [27] that simultaneously target women with prior GDM, their family and the health care system will be effective at reducing T2D risk by lowering body mass index (BMI) and increasing the quality of life.

## Methods/Design

This protocol follows the guidance for protocol development and reporting described in the Standard Protocol Items: Recommendations for Interventional Trials (SPIRIT) 2013 statement (*see* Additional file 1).

### Design

**Fig. 1:**
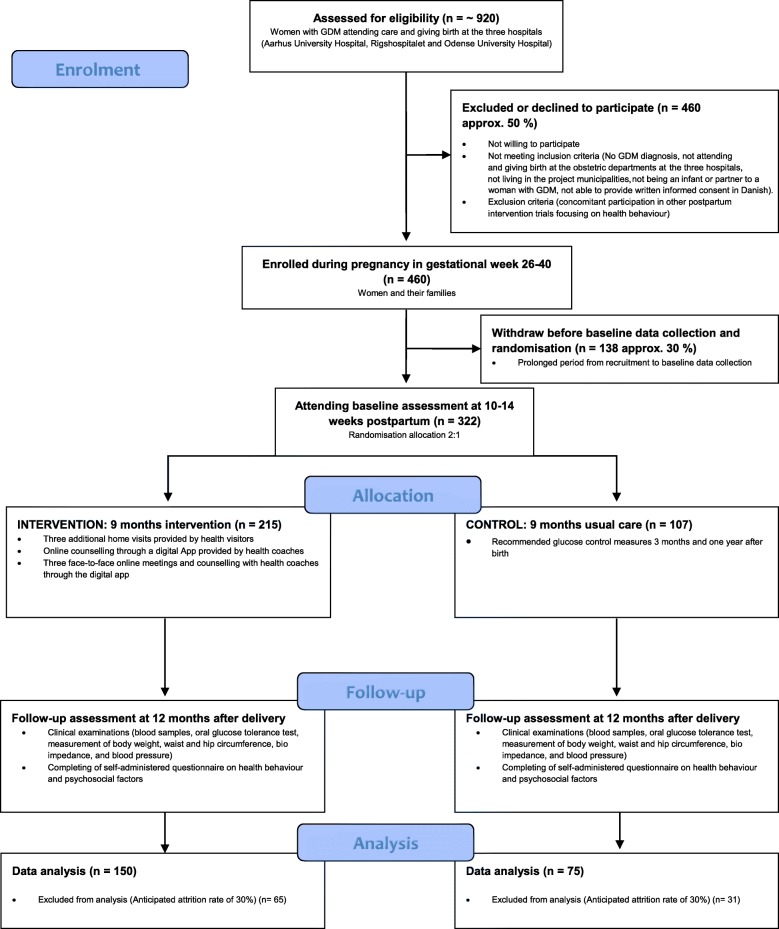
CONSORT diagram for Face-it showing participant flow through the phases of the randomised controlled trial

The study is a superiority RCT with two arms comparing a complex intervention with usual care to reduce BMI. The allocation ratio will be 2:1 to the intervention (2/3 of participants) or usual care (1/3 of participants) groups. The imbalance was pragmatically chosen to ensure the smooth running of the intervention programme, including ensuring a critical mass for running the intervention activities. The CONSORT diagram illustrates the design (Fig. 1).

### Patient and public involvement (PPI)

Women with prior GDM and their partners, as well as health professionals, such as health visitors (nurses specialised in postnatal, maternal and child health), obstetricians, endocrinologists, and dieticians, worked with us to develop and refine the intervention content and delivery methods. PPI representatives also contributed to the selection of core outcome measures, validation of the questionnaire and the optimisation of all written materials to be disseminated to their peers in connection with the study. The Face-it study is supported by an advisory group with PPI representatives, who will provide ongoing input and feedback throughout the duration of the study.

### Settings and recruitment

In Denmark, all women with GDM are managed in a hospital setting by multidisciplinary teams including physicians, dieticians, nurses and midwives. Women with GDM who are candidates for participation will be recruited from obstetric departments in three university hospitals: Aarhus University Hospital, Rigshospitalet and Odense University Hospital. These three hospitals are located in the three largest cities in Denmark (Aarhus, Copenhagen and Odense). The participants will be identified, assessed for eligibility and approached by a health care professional at approximately 24–40 weeks of pregnancy and invited to participate in the study. Candidates for participation will be able to ask questions and have time to consider their decision before providing written informed consent. If the candidate wishes to participate in the study, contact information and expected delivery date will be recorded by the recruiting health care professional. Participants will be contacted postpartum and invited to the baseline data collection visit, which will take place at a hospital clinic in each of the three cities at 10–14 weeks after delivery. Participants will also be asked for consent for the storage of blood samples in biobanks for future ancillary studies. Finally, we will ask for consent to collect data from the women’s medical records to obtain information on conditions during pregnancy and birth as well as the anthropometric measurements of the infant taken at birth.

Both participants and those who decline study participation will be asked for consent to extract data from their medical record up to 4 months after delivery. This will allow us to describe and compare the characteristics of participants and non-participants.

If the woman’s partner does not attend the consultation where recruitment takes place, the woman will be asked to convey information and material on the study to her partner. The partner will subsequently have the opportunity to attend a face-to-face or telephone meeting with the recruiting health care professional. Partners who agree to participate will also be invited to the baseline data collection visit.

### Eligibility criteria

Women with a GDM diagnosis when attending or giving birth at one of the three recruiting hospitals will be eligible to participate. According to Danish guidelines, GDM is diagnosed when, following a 75 g oral glucose tolerance test (OGTT), the 2 h value is ≥ 9.0 mmol/l (venous plasma or capillary blood). In Denmark, a risk-factor based screening approach is followed. Hence, an OGTT is performed at 24–28 gestational weeks if the woman has maternal pre-pregnancy BMI ≥ 27 kg/m^2^, polycystic ovarian syndrome (PCOS), a family history of diabetes, a multiple pregnancy or previously has given birth to a baby weighing ≥ 4500 g. If she has more than one of these risk factors *or* has a prior GDM diagnosis *or* glucosuria, she will also be offered testing in early pregnancy, i.e., 14–20 gestational weeks.

Women taking part in the trial also have to 1) provide personal contact details; 2) live in either Aarhus, Copenhagen or Odense municipalities; 3) participate in the physical examinations; 4) complete a questionnaire on behavioural and psychosocial measures; 5) allow the research team access to their medical records; 6) be followed for approximately one year; 7) allow the research team to contact and invite the woman’s partner to participate in the study; and 8) be able to understand and provide written informed consent in Danish.

In addition, partners and new-born offspring of participating women will also be eligible to join the trial if the following criteria are met. The partners should 1) be a partner to a woman participating in the Face-it trial, 2) provide personal contact details, 3) participate in the physical examinations, 4) complete a questionnaire on behavioural and psychosocial measures, 5) be followed for approximately 1 year and 6) understand and provide written informed consent in Danish. The partner is not required to live in the same household as the participating woman. Offspring will be included if the mother provides written informed consent on behalf of her child. Trial eligibility for women and their offspring does not depend on the participation of a partner.

The intervention and research questionnaires will only be available in Danish. Participants who are not able to provide informed consent in Danish will therefore be excluded. Furthermore, women who are not able to understand the informed consent and/or study procedures, as well as women already participating in other postpartum interventions, will be excluded. Based on the results of the OGTT performed at baseline, women who at this time are diagnosed with overt diabetes will not be eligible for the RCT and will instead be referred to their local endocrinology department or GP. Overt diabetes will be defined as fasting venous plasma glucose ≥7.0 mmol/l and/or a 2 h 75 g OGTT venous plasma glucose ≥11.1 mmol/l [28].

### Primary outcome

Although the ultimate outcome of the study is reduction in maternal T2D risk, based on previous studies (e.g., [29–31]), we do not anticipate seeing such effects at 12 months after delivery. Therefore, the primary outcome is a reduction in BMI from baseline to 12 months after delivery among women with prior GDM. Existing studies have established BMI as a strong predictor of incident T2D in both high-risk and population-based studies [32], including in women with prior GDM [33]. We hypothesise that, at 12 months after delivery, women with prior GDM randomised to the Face-it intervention will show significantly larger reductions in BMI compared to those receiving usual care and that such a reduction in BMI is a marker for reduced diabetes risk.

### Secondary outcomes

We will carry out assessments related to a number of secondary outcomes, including other anthropometric measurements (e.g., body composition), blood tests, OGTT, blood pressure, self-reported diet and physical activity, breastfeeding, quality of life, health literacy, physical and mental health status, risk perception and social support. These measurements will be collected at baseline and 12 months after delivery.

### Sample size calculations

Calculation of the projected sample size is based on individual changes in BMI among women with prior GDM after 12 months of follow-up. The mean changes are compared between the intervention and control group. The expected size of the change and their variation is based on previous intervention studies [7, 10]. We expect the mean change in the intervention group to differ by − 1.0 kg/m^2^ relative to the control group. The standard deviation of individual changes after 12 months is expected to be 2.5 kg/m^2^.

Based on a 2:1 randomisation procedure, a power of at least 80% and type 1 error of 5% (two-sided), a sample size of 225 will be required to detect such a difference in BMI. Assuming 30% of participants will be lost between baseline and follow-up, we will need to include 322 women at baseline in the study. Because of the relatively long period (10–30 weeks) between recruitment and baseline, we also assume that 30% of those agreeing to participate will withdraw before baseline data collection and randomisation. We therefore need to recruit a total of 460 women.

The total annual number of women with GDM at the three hospitals is approximately 700 (AUH: 200, OUH: 330, and RH: 170). We expect 50–60% of women with GDM will be eligible and agree to participate. Completion of enrolment into the RCT is therefore expected to take 1–2 years.

To ensure adequate participant enrolment into the study, we will provide ongoing training and supervision of recruiting health care professionals. The health care professionals conducting the recruitment are in contact with the target group as part of standard GDM care during pregnancy. Findings from the co-production phase suggest that this will increase the women’s/families’ motivation to participate. Furthermore, marketing materials in the form of posters and flyers have been produced. We will optimise retention by ensuring that study visits are carefully planned and scheduled to minimise inconvenience for the study participants. Moreover, to ensure high participation at the baseline visit, an email will be sent to the families 4–6 weeks before the baseline visit, and an SMS reminder will be sent 24 h prior to the baseline visit. Finally, remuneration in the form of a maternity gift will be offered to participants after completion of the first study visit.

### Study duration

Recruitment into the trial commenced in May 2019. The first randomisation took place in August 2019. The primary endpoint will be captured after the end of the intervention, i.e., 12 months after delivery. Enrolment into the study is likely to be complete by August 2021. The study will therefore end by January 2023, with an overall study duration of 43 months.

### Randomisation and blinding

Randomisation to either the intervention or usual care group will occur immediately after baseline data collection. An independent statistician (Henrik Støvring) has generated the randomisation procedure, and the allocation sequence will be implemented using the REDCap (Research Electronic Data Capture) electronic system. Randomisation will be in random blocks of 6/9/12/15, with a separate randomisation at each of the three recruitment locations. This ensures that allocation to intervention or usual care is unpredictable for recruiters and that the cumulative ratio of participants included to intervention vs. usual care is close to 2:1 throughout the inclusion period at each hospital. When more than 100 women have been included at a hospital, 65% to 68% of participants will have been allocated to the intervention. Allocation will be concealed from both the participant and the investigators until participation has been accepted, eligibility confirmed, and baseline data collected. Neither participants nor the investigators will be blinded to the participants’ allocation status after this point. However, the analysts will be blinded to the allocation groups. Both the intervention and usual care group will be part of the evaluation of the Face-it trial and therefore invited to baseline and follow-up clinical examinations.

### Components of the complex intervention

The development of the intervention followed the UK Medical Research Council framework for the development and evaluation of complex interventions [27]. We carried out a needs-assessment drawing on the co-production approach described by Hawkins and colleagues [16] to ensure a careful tailoring of the intervention. The theoretical framework and development framework process for Face-it will be described elsewhere.

The complex health promotion intervention consists of three major components: i) active involvement of health visitors in addition to usual care, ii) digital health technology, iii) and a structured cross-sectoral communication system in the health care system. The intervention will begin at 3 months (after the baseline data collection, i.e., at 10–14 weeks postpartum), and will continue until 12 months after delivery.

The first component of the intervention will focus on the health visitor as a source of support, information (e.g., on disease risk and prevention) and motivation to engage in healthy behaviour change. Health visitors are nurses, with further specialisation in postnatal and child health, who carry out home visits to families in the postnatal period as a public health service in Denmark. The general health visitors seek to promote health and wellbeing of the infant and family. In the Face-it intervention, the families will receive three additional visits by a health visitor further trained in GDM and prevention of T2D. The visits cover five different themes: 1) GDM and risk/prevention of T2D; 2) daily routines; 3) food and meals; 4) exercise/movement; and 5) family, friends and network. The health visitors will also help participants navigate the health care system, thus increasing health literacy, and facilitate the active involvement of the partner to increase positive family dynamics and social support around health behaviour change.

The second component of the Face-it intervention is the LIVA digital platform (app), which combines digital health behaviour coaching and goal setting with virtual support groups. The platform is already being used by persons with T2D in 10 Danish municipalities and in a number of UK settings. For the Face-it study, the platform has been tailored to women with prior GDM and their partners, e.g., by including additional goal setting options such as breastfeeding. It also provides information on local health promotion events and materials aimed at motivating and supporting women and their families to engage in healthy behaviours. Both women and their partners will be offered individual personalised coaching by health coaches during the intervention period. After allocation to the intervention group, participants will be contacted by a health coach in order to initiate the digital coaching component. The standard regimen offered is weekly coaching for the first 3 months of the intervention; coaching every second week the following 3 months; and coaching once every month for the last 3 months of the intervention. However, the frequency of contacts will be flexible and largely depend on the needs and wishes of the participants. The health coaches will have various professional backgrounds (nursing, public health, etc.) and coaching experience. They will receive specific training related to GDM, T2D risk and digital health coaching for this project by the research team and collaborators.

The third and final component of the intervention focuses on strengthening communication and coordination between the various health care professionals involved in care for the target group, i.e. obstetric departments, GPs and health visitors. Currently, the woman’s GP receives a discharge letter from the obstetric ward. In the Face-it intervention, we add discharge communication to the health visitor. This component also entails health visitors and health coaches reminding and encouraging participating women with prior GDM to book and attend the recommended regular glucose testing and counselling with their GP.

Participants in the usual care group will receive usual practice, including recommended glucose control measures at 3 months and 12 months after delivery and usual care from a health visitor. Participants will receive advice about maintaining a healthy lifestyle via national recommendations from the Danish Health and Medicines Authority. Participants in the control group will be invited to participate in the health examination at baseline and follow-up and receive information about their own health when attending the two clinical examinations.

### Adherence

Participants randomised to the Face-it intervention will receive the full intervention. The minimum intervention dose has been pre-defined prior to intervention roll-out. Adherence to the intervention will be monitored through data obtained from the LIVA digital platform and collected by the health visitors. From the LIVA digital platform, information about the kind of goals and how frequently the participants have registered their achievement will be extracted as will the number of contacts with their health coach. Adherence to the health visitor component will be assessed using a self-administered questionnaire on the number and duration of home visits as well as the topics covered during those visits.

### Data collection

We will collect information from medical and birth records for all women with prior GDM participating in the trial and from women who withdraw or decline to participate (following informed consent for access to medical records). Data will be used to 1) assess trial eligibility of potential participants, 2) measure participation rates, 3) evaluate the characteristics of those declining or withdrawing participation and 4) provide obstetric information on trial participants.

Measurements will be conducted at 10–14 weeks postpartum (baseline) and 12 months after delivery (follow-up). Data collection visits will take place in the morning and the participants (not offspring) must fast before both visits (overnight fast > 8 h). *See* Table 1 for details.

- Anthropometric measures: To assess changes in BMI, height and body weight will be measured with the participant barefoot and wearing light indoor clothes. Height will only be measured at baseline. Waist circumference will be measured halfway between the lowest point of the costal margin and highest point of the iliac crest. Hip circumference will be measured at the level of the greater femoral trochanter. Both will be measured to the nearest 0.5 cm. Body fat percent will be measured using a non-invasive body composition analysis (lnBody 270) that provides a detailed distribution of the participant’s weight in terms of muscle, fat, and water (bioimpedance).
- Blood pressure will be measured with the participant in a sitting position after a minimum 15 min of rest and with an average of three readings taken at 2-min intervals. Similar devices (Microlife BP A3L comfort) will be used at all three sites.
- Biochemical measurements: Blood samples will be drawn after an overnight fast and will include measures of glucose (fasting glucose and HbA1c), insulin, lipids and plasma gamma-glutamyltransferase (GGT) in both women with prior GDM and their partners. We measure GGT to be able to calculate the fatty liver index (FLI), which is an algorithm based on waist circumference, BMI, triglyceride and GGT. FLI has been found to be predictive for non-alcoholic fatty liver disease, which is a strong risk factor for T2D and cardiovascular disease. A 75 g OGTT will also be performed in women with prior GDM with measurements taken at 30 and 120 min. A bio-bank for future research, including storage and registration of blood samples, has been approved, allowing for further biochemical investigations within this area of research in the future.
- Questionnaires: women with prior GDM and their partners will be asked to fill out a self-administered, electronic questionnaire. The questionnaires will assess self-reported health behaviours (dietary, physical activity, sedentary behaviour, sleep, smoking, alcohol, and breastfeeding), quality of life, health literacy, demographic information, socio-economic information, obstetric/medical history including current use of glucose-lowering medication, mental health and wellbeing as well as various psychosocial factors, including risk perception and social support (see Table 1 for details).

**Table 1:** Data collection procedures in the Face-it Trial

Variable	Measurement	Participant	T0	T1	T2
Anthropometry	Height, weight and calculation of BMI	W		●	● (weight only)
		P		●	● (weight only)
		B	●		●
	Waist circumference, hip circumference, % body fat	W		●	●
		P		●	●
	Abdominal circumference, head circumference	B	●		●
Blood pressure	Systolic and diastolic blood pressure	W		●	●
		P		●	●
Glucose and Insulin	Fasting glucose, fasting insulin, HbA1c, calculations of HOMA-IR and HOMA-β	W		●	●
		P		●	●
	Glucose and insulin at 30 and 120 min 75 g OGTT	W		●	●
Lipids	Total cholesterol, triglycerides, HDL, LDL	W		●	●
		P		●	●
Plasma gamma-glutamyl transferase		W		●	●
		P		●	●
Demographics, socio-economics, medical history	Self-constructed questionnaire	W		●	●
		P		●	●
Obstetric history	Self-constructed questionnaire	W	●	●	
Quality of life	Short Form Health Survey (SF-12v2) [34]	W		●	●
		P		●	●
Self-perceived health	Using the question ‘in general, would you say that your health is excellent, very good, good, fair, or poor?’	W		●	●
		P		●	●
Mental health and wellbeing	Perceived Stress Scale (PSS) [35], General Anxiety Disorder scale (GAD7) [36], The WHO-Five Wellbeing Index (WHO-5) [37]	W		●	●
		P		●	●
Diet, physical activity, sleep, alcohol and smoking	Dietary Quality Score [38], International Physical Activity Questionnaire (IPAQ) short form [39], Karolinska Sleep Questionnaire (KSQ) [40, 41], Self-constructed questionnaires	W		●	●
		P		●	●
Breastfeeding	Self-constructed and questions from the Infant Feeding Practices Study II [42], Nilsson et al. 2017 [43], and the Danish National Birth Cohort [44]	W		●	●
Health literacy	Health Literacy Questionnaire (HLQ) [45, 46]	W		●	●
		P		●	●
Risk perception and knowledge about diabetes	Risk Perception Survey for Developing Diabetes (RPS-DD) [47, 48] adapted to Danish context	W		●	●
		P		●	●
Self-efficacy	Maindal 2009 [49]	W		●	●
		P		●	●
Perceived quality of health care services	Health Care Climate Questionnaire (HCCQ) [50] adapted to GDM	W		●	
Exercise Self-regulation and motivation	Exercise Self-regulation Questionnaire (TSRQ-E) [51] modified	W		●	●
		P		●	●
Social support	Perceived Social Support [52]; Social Support for Diet and Exercise Behaviours [53]	W		●	●
		P		●	●
Family Functioning	McMaster Family Functioning Scale, General and Roles domains [54]	W		●	●
		P		●	●

*W* woman with prior GDM, *P* partner of the participant, *B* baby of the participant,

*T0* data collected from medical birth record, *T1* data collected at baseline (10–14 weeks after delivery), *T2* data collected at follow-up (12 months after delivery)

### Planned data analysis

The primary analysis will be based on the intention-to-treat approach; thus, we will include all participants in their original randomisation group regardless adherence to the intervention. Descriptive statistical analyses will be performed using the chi squared test, Fisher’s exact test (if expected cell count < 5), t tests (normally distributed data) or Mann-Whitney U test (non-normally distributed data) where appropriate. We will use two-sided significance tests at the 5% level. We will use regression models to adjust for confounding variables if necessary, i.e., if randomisation has not ensured similar or equivalent distribution of baseline characteristics in the two randomisation groups. Specifically, we will investigate if potential use of glucose-lowering medications may have influenced the results. We will include a random effect for each hospital, so that the effect can be estimated by comparison within hospitals. This will improve the precision of the estimate compared to an analysis comparing treatment arms across hospitals [55]. The sample size calculation presented above is therefore conservative compared to the proposed analysis. We have not accounted for this expected gain in precision in the power analysis as we do not have relevant and credible information on variation between the hospitals.

Participants will be free to withdraw from the trial at any time without giving a reason. Investigators may also withdraw participants from the study due to safety concerns or non-compliance with the protocol. Participants who are withdrawn from the study will not be replaced. Data collected prior to withdrawal/drop-out will be included in some analyses, e.g., baseline results. The effect evaluation requires data collected at follow-up to assess the change in BMI (and secondary outcomes). We will investigate/seek to overcome attrition bias by performing both a per-protocol analysis and an analysis based on worst case scenario and/or imputation. In addition, we will be able to follow participants, including drop-outs, in the Danish health registries, which will provide additional information on whether participants are diagnosed with T2D in the long run.

### Ethical considerations

This study will be carried out in accordance with the Declaration of Helsinki. Ethical approval for the study has been granted by The Regional Scientific Ethics Committee of the Capital Region, Danish National Committee on Health Research Ethics (approval number: H-18056033). Any protocol amendments will be reported and submitted to the Ethics Committee.

Anonymity and confidentiality of participants will be ensured by assigning a study ID number to all participants (both women with prior GDM, partners and offspring). Informed consent will be obtained from all participants.

### Data monitoring and management

All data will be entered and stored in the Electronic Data Capture system, REDCap. This is compliant with EU General Data Protection Regulation (GDPR) and Good Clinical Practice guidelines [56, 57]. The study adheres to all GDPR-regulations and the Danish Act on supplementary provision to the regulation on the protection of natural persons with regard to the processing of personal data and on free movement of such data. All health-related and sensitive personal data (blood samples, etc.) will be depersonalised. A trial ID will be assigned to all participants and personal information will be stored securely and separately.

Data will be entered directly into electronic Case Report Forms (CRF) using REDCap. Data from the women’s medical records will be entered by a project-affiliated health care professional at the obstetric ward. At baseline and follow-up, questionnaire data will be filled out electronically and entered directly into REDCap by the women and their partners. All other data will be entered by the investigator responsible for the baseline or follow-up examination. The REDCap data collection instruments for this project have been designed with restrictions, warning systems, instructions, piping and branching to minimize the risk of data entry errors.

If access to the REDCap system is not possible, data will be collected on paper CRF and entered into REDCap when access has been restored.

Quality checks and verification of entered data will be carried out regularly by the research team both at the aggregated and individual participant level. We will check for missing data, internal consistency, range for data values and obvious errors. Once a data collection form has been checked and verified, it will be locked from further editing. A detailed internal data management plan in Danish is being developed by the research team.

Monitoring will also be performed regularly by an experienced external researcher, who will check adherence to study protocol and completeness of the data collection forms.

We anticipate collecting data on all participants regardless of adherence to intervention protocols. All participants, including participants lost to follow-up, will be followed in national registries for the development of T2D beyond the 1-year follow-up point.

### Dissemination plans

Results will be disseminated in international and national peer-reviewed scientific journals and at local and international conferences. We also plan to share results with the public through printed and electronic mass media, e.g., via press releases, the project website (www.Faceit-info.dk), newsletters and stakeholder meetings. The detailed dissemination plan will be refined by the study leadership. Authorship will be based on the Vancouver guidelines.

## Discussion and implications

This study protocol describes the first RCT to examine the effectiveness of a complex health promotion behavioural intervention to reduce T2D risk and improve wellbeing in Danish women with prior GDM and their families. Previous international studies seeking to reduce the risk of T2D following GDM have shown varying results, probably reflecting both the heterogeneity in GDM populations as well as the design and implementation of the various interventions. A key feature of the Face-it intervention is the extensive development and co-production design of the intervention with the target group. Such participatory approaches improve the ownership and relevance of the intervention for the target group [58].

Previous intervention studies aimed at T2D prevention in women with prior GDM have struggled with recruitment, engagement and retention rates [59–61]. By involving the target group in the design of the study, the Face-it intervention is tailored to the needs and challenges of participating families and is feasible in the context of their everyday life. We hope this will encourage a positive response to the intervention and promote high rates of recruitment and engagement.

The Face-it intervention not only focuses on physical health but addresses a broader perspective, including mental and social wellbeing. Gilbert et al. argue that the integration of psychosocial wellbeing (e.g., social support) in interventions seeking to reduce the adverse impacts of GDM is important given the documented interaction with physical activity and dietary choice [62]. Likewise, in their recent systematic review, Buelo and colleagues highlight the importance of psychosocial factors, such as social and community support, and addressing everyday barriers (e.g., having time to exercise) for physical activity interventions for women with prior GDM to be effective [63]. Consequently, we expect the Face-it intervention to be a better ‘fit’ for the target group, increasing the likelihood of engagement and behaviour change compared to previous interventions.

With increasing rates of T2D and the substantial costs associated with the condition both in financial and personal terms [64, 65], a substantial need exists for identifying effective T2D prevention approaches. We also need to develop, improve and evaluate such interventions. While the current study has a 1-year follow-up, we plan to follow the families for a longer period through linkages with Danish registries and potentially with clinical investigations and questionnaires. In the study, BMI was selected as the primary outcome. While BMI is a well-established strong predictor of T2D development, it could be argued that a measure of glucose would be a more obvious choice of primary outcome. However, in their meta-analysis of existing lifestyle interventions aimed at T2D prevention in women with prior GDM, Goveia et al. found no effect on glucose measures but did find a moderate reduction in BMI [9]. As argued by the authors, while the observed effects are small, a modest change in anthropometric measures within a short time in relatively young women may still have a substantial impact on the long-term risk of T2D [9]. Thus, based on prior intervention studies targeting women in the first years(s) after their GDM affected pregnancy, we concluded that the existing evidence base for changing BMI in this group was relevant and stronger in terms of providing us with necessary information upon which we could base our sample size calculation. Both BMI and measures of glucose have been identified as core outcomes to be measured in intervention studies targeting women with prior GDM in recent core outcome set studies [66, 67].

Importantly, this study will not solely assess the effectiveness of the intervention with respect to risk factors, but will also measure a broad range of health, quality of life and social outcomes. Associated studies will also capture partner and offspring outcomes. Furthermore, the associated costs will be established, and the effectiveness evaluation will be coupled with health economic evaluations of the Face-it intervention. In an auxiliary feasibility project, we are also investigating which modifications are required to offer the intervention to women with prior GDM in Denmark from ethnic minority backgrounds with limited Danish language skills (who are currently ineligible for participation in the Face-it trial). In addition, we will carry out process evaluations at the family level and within the health care system, which will help shed light on why/why not the intervention is/is not effective. This will also include an assessment of the penetration and participation of the study and whether those women participating in the study are different than women with GDM in Denmark in general. In addition, while we exclude potential participants with overt diabetes at baseline; we do not exclude participants taking glucose-lowering medication, e.g., metformin for PCOS. This may be a limitation of our study if the randomisation procedure fails to ensure an equal distribution in the two groups. Furthermore, we measure health behaviours using questionnaires. Self-reported measures entail risk of reduced accuracy. Therefore, in the construction of the questionnaire, we have followed recommendations on how to maximise self-reported information [68], including relying on validated scales and questionnaires; phrasing questions in a way that would minimise socially desirable responses and pilot testing the questionnaire to ensure it is fully understandable. In addition, we are planning to include objective measures (accelerometry) for physical activity in a subgroup of our sample in order to extent and validate our self-reported measures. This comprehensive evaluation is expected to contribute much needed evidence to support policy-makers in making balanced decisions about how to promote health for families to reduce their risk of T2D and associated conditions, as well as for similar preventive services conducted in a close cross-sectional collaboration.

## Trial status

The Face-it trial was registered on clinicaltrials.gov (NCT03997773) on 25 June 2019. Recruitment of participants commenced in May 2019. The first participant attended baseline examination in August 2019. Recruitment is expected to be completed by August 2021. This is protocol version 1, dated 11 July 2019.

## Supplementary information

**Additional file 1.** SPIRIT Checklist.

## Data Availability

Not applicable.

## Abbreviations

BMI: body mass index
CRF: case report form
DPP: Diabetes Prevention Program
GDM: gestational diabetes mellitus
GDPR: General Data Protection Regulation
GGT: gamma-glutamyl transferase
OGTT: oral glucose tolerance test
PCOS: polycystic ovarian syndrome
PPI: patient and public involvement
RCT: randomised controlled trial
REDCap: Research Electronic Data Capture
T2D: type 2 diabetes mellitus
